# Population health management in Saudi Arabia: A narrative review of system undergoing reform

**DOI:** 10.1016/j.dialog.2026.100292

**Published:** 2026-03-16

**Authors:** Khalid Alkhurayji, Abdallah Alsuhaimi, Sultan Aldakhil, Dalal Alshathri

**Affiliations:** a Research, Statistics, and Information Department, Saudi Central Board for Accreditation of Healthcare Institutions, Riyadh, Saudi Arabia; b Executive Director of Standards, Saudi Central Board for Accreditation of Healthcare Institutions, Riyadh, Saudi Arabia; c College of Dentistry, King Saud Bin Abdulaziz University for Health Sciences, Riyadh, Saudi Arabia; d Department of Restorative Sciences, Adams School of Dentistry, University of North Carolina at Chapel Hill, Chapel Hill, NC, USA

**Keywords:** Population health, Healthcare system, Public health, Healthcare services

## Abstract

Background the Saudi Arabian healthcare system is undergoing transformation towards 2030 vision, with increase recognition of Population Health Management (PHM) as a critical approach to enhance health system efficiency, health outcome, and preventing the burden of chronic diseases. Despite the implementation, integration, and policies intentions of PHM in Saudi Arabia, there is a need to inform ongoing transformation through consolidated and contextual understanding of PHM Methods This narrative review synthesis published literature examining PHM in the context of Saudi Arabia. relevant studies were identified through multiple databases and search engine including PubMed, Web of Science, and Google Scholar. Thematic analysis was conducted to analyze the content and generate themes across the included studies Results Five emerging themes were reported in terms of Healthcare services, policies and regulations, economics, e-health, and health insurance across 34 studies. The healthcare services in Saudi Arabia are reported to be facing many obstacles that have to be addressed to provide the population with optimal services that can follow the revolutionary vision of Saudi Arabia. Among these challenges, insufficient population education and engagement were consistently identified as major barriers to effective PHM implementation. Conclusion The transformation plan of Saudi Arabia is promising, yet the success of these reforms depends on the population. Hence, more focus is mandated on strengthening population engagement, system integration, and governance capacity. This narrative review underscores the need to focus on population-centered strategies and to translate structural reforms into measurable health gains and sustainable system performance.

## 1 Introduction

Globally, the Population Health management (PHM) emerged as a central strategy to improve healthier lives for the population, reduce the financial healthcare costs, in addition to addressing the issues related to inequalities due to epidemiological transitioning, the rise of the chronic diseases burden, and demographical shifts. Healthcare systems worldwide strive to adapt PHM approaches in order to mitigate risks, improve data integration, and coordinate services delivered across the continuum of care. Despite the contribution in this field, persistent challenges remained in terms of governance, workforce, and financing management [1], [2], [3].

Population health management refers to the strategic approach taken by healthcare systems and organizations to improve the overall health outcomes of a given population. This represents a broader concept than disease management, as it seeks to address not only individual chronic conditions but also comprehensive care coordination for individuals belonging to high-risk groups. [4], [5]. As healthcare systems in Saudi Arabia undergo reform, the focus on population health management has become increasingly important. Saudi Arabia, through its Vision 2030 roadmap, has recognized the need to improve the quality of healthcare in the country [6], [7]. This strategy includes expanding the private sector's role in delivering and improving primary healthcare services. At the same time, there is a growing demand for healthcare services, such as periodic check-ups of risk factors and early disease detection [8]. This ongoing healthcare reform underscores the growing importance of population health management in Saudi Arabia. By implementing population health management strategies, healthcare organizations in Saudi Arabia can effectively manage the health of their entire population—not only those who actively seek care. This approach can lead to improved prevention and care for chronic diseases, but it also presents challenges, such as a shortage of healthcare resources. According to Chowdhury, Mok and Leenen [6] study, the primary healthcare system in Saudi Arabia is being reformed as a key contribution towards the Vision 2030 goals. Involving community pharmacies in providing public health services is seen as a potential solution to add value to the primary healthcare system. In addition, the healthcare reform process in Saudi Arabia highlights the importance of enhancing the quality of care through the development of a well-qualified nursing workforce and effective management practices [9]. These human resources are fundamental to population health management, as evidence demonstrates that healthcare worker satisfaction and competence directly influence the quality and accessibility of population-level services [10]. Furthermore, a comprehensive approach that addresses both the healthcare delivery system and the work environment of healthcare employees is essential for sustainable population health outcomes. Prior research examined the evidence to provide a system-level overview regarding PHM. However, certain literature gaps remained persistent, which motivates conducting this research to consolidate existing evidence, identify key challenges, and support healthcare leaders and policymakers in aligning PHM efforts with the goals of Vision 2030. Given the challenges and opportunities associated with healthcare reform in Saudi Arabia, PHM emerges as a critical strategy for enhancing the overall health outcomes of the population. Ultimately, it serves as a cornerstone of the nation's healthcare transformation efforts, supporting the goal of improving care quality and meeting the evolving needs of a growing population. Despite ongoing efforts, the Saudi healthcare system faces increasing pressures due to rising prevalence of chronic diseases such as cardiovascular disease and diabetes, as well as fragmentation in healthcare service delivery. Together, these challenges underscore the need for effective PHM. Evidence from a Saudi tertiary care hospital demonstrates suboptimal glycemic and cardiovascular risk control among patients with type 2 diabetes, highlighting the urgent need for comprehensive population-level interventions to address these chronic disease challenges [11]. Nonetheless, the limited descriptive reports and sector-specific studies making it difficult to understand how PHM implemented, what challenges persist, and how these efforts can be effectively aligned to improve population health outcomes. As a results, further research in these area are need to explore and address the literature evidence and the primary contribution of this review lies in the recommendations provided in terms of PHM in Saudi Arabia. Therefore, this narrative review aims to synthesize recent literature on population health management in Saudi Arabia to identify key challenges and opportunities and provide recommendations aligned with Vision 2030. To achieve this objective, we employed a systematic approach to literature identification and thematic analysis, as described in the following section.

## 2 Materials and methods

### 2.1 Search strategy

Through multiple research database engines (PubMed, Google Scholar, and Web of Science) between 2020 and 2024, using the terms (“administration” OR “management”), in combination with (“public health” OR “epidemiology “OR “Population Health”) AND “Saudi Arabia”. Searches were also conducted through the grey literature using Google Scholar and WHO IRIS. Only peer-reviewed articles were included, while non-peer-reviewed publications were excluded from the review. A hermeneutic approach was adopted to address the research question [12] It is an approach that is suitable for questions requiring clarification and insight through the literature of transdisciplinary synthesis [13]. Moreover, the PICO (Population, Intervention, Comparison, and Outcome) model was used to refine the research question, shaping the focus and structure of Table 1 [14].

**Table 1:** Population, intervention, comparison, and outcome (PICO) model.

Items	Terms
Population (P)	Patients in Saudi Arabia
Intervention (I)	Health Management
Comparison (C)	Not Applicable
Outcome (O)	Recommendation on health management

What is the current literature on population health management in Saudi Arabia?

Relevant articles were identified through title and abstract screening, while citation-tracking software was used to locate additional sources as the analysis evolved, consistent with the hermeneutic review approach, which emphasizes iterative mapping and classification of literature. EndNote software (Clarivate Analytics) (Version 20) was used during the citation-tracking process.

### 2.2 Inclusion criteria

Included studies must be located in Saudi Arabia, population-wide, and conducted between 2020 and 2024. The timeframe of 2020–2024 was selected to capture recent evidence reflecting the current state of healthcare reform in SA, particularly following the acceleration of Vision 2030 implementation initiatives and the impact of the COVID-19 pandemic on the healthcare system.

### 2.3 Exclusion criteria

Studies were excluded if they were published in languages other than Arabic or English, or if they were non-peer-reviewed articles.

### 2.4 Analysis of results

The six steps of thematic analysis were used across the studies, following the protocol of Braun and Clarke [15]. The researchers first familiarized themselves with the data through repeatedly reading from the included studies in order to gain a holistic understanding of the context and content. Followed by initial coding, which was generated systematically for PHM. The following steps involved examining the coded data to identify patterns and themes from the recurring content, resulting in the development of the preliminary themes, which were reviewed and refined across other themes by ensuring consistency, coherence, and distinctions with the overall data set. Each theme was defined and named to reflect the core component, meaning, and relevance to the objectives of the study. Finally, the themes' syntheses were reported in a structured narrative.

**Fig. 1:**
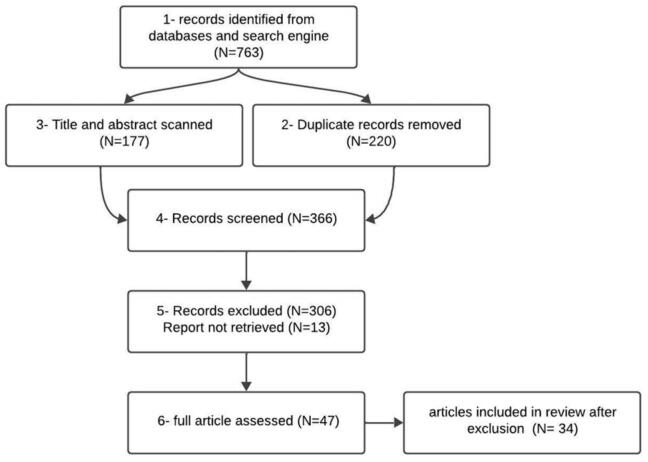
Flow chart of the studies selection process.

The study's results explored the population health management evidence in the literature with the use of a broad search strategy. Fig. 1 presents the flow chart of the literature search and study selection process, including records identified, screened, excluded, and included in the final synthesis.

## 3 Discussion

This narrative review synthesized evidence from 34 studies published between 2020 and 2024 examining various aspects of population health management in Saudi Arabia. The evidence base reflects growing research interest in digital health solutions (7 studies), healthcare service delivery and efficiency (19 studies), and policy frameworks (5 studies), with relatively fewer studies examining economic factors and health insurance mechanisms (5 studies each). Most of the included studies were observational or descriptive in nature, with limited intervention or implementation research. Geographically, most studies focused on urban centers or employed national-level data, with fewer studies specifically examining rural or underserved populations.

Thematic analysis of this evidence revealed five major themes that structure the discussion below: economy, e-health, policies and regulations, health insurance, and healthcare services. These themes represent the key domains through which PHM is being conceptualized, implemented, and evaluated within the Saudi healthcare context.

**Fig. 2:**
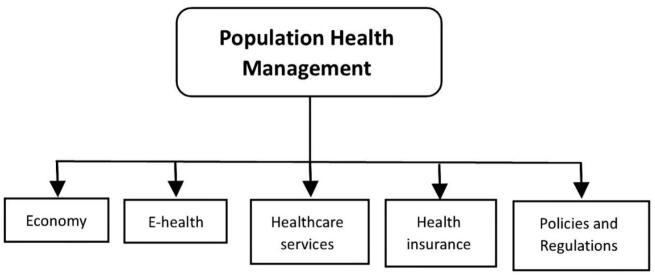
Population health management themes.

The results of the thematic analysis after gathering the selected data, critically crafting the codes, and finally establishing themes. Resulting in five outlines, economics, e-health, policies and regulations, health insurance, and healthcare services were the outcome of the analysis.(Fig. 2). Table 2 summarizes the recommendations of the reviewed studies within each of these themes, highlighting the main focus and policy implications discussed in the literature.

**Table 2:** Studies recommendations in each theme.

Recommendation	Theme	SOURCE (AUTHOR, DATE)
Policymakers must recognize and address these variations when formulating and implementing digital health policies.	e health	(Alhodaib et al. 2021(
Regular evaluation is essential to monitor users' evolving needs and update the app accordingly.	e health	(Alanzi et al. 2022)
The Ministry of Health should raise public awareness of the app and its benefits.	e health	(Aldhahir et al. 2022)
It is essential for the Ministry of Health to promote its digital applications through the portal and enhance public awareness of their use. Accordingly, improving the usability and design of these applications requires a careful assessment of the needs and preferences of the Saudi population.	e health	(Al-Rayes et al. 2023)
The success rate of EHR implementation is often influenced by the scale of the project. Key factors such as staff training, technical support, legal considerations, and organizational workflows must be adequately addressed to ensure effective implementation.	e health	(Alzghaibi et al. 2022)
Integrating the gig economy into eHealth systems may enhance the efficiency and effectiveness of eHealth operations.	Economic	(Alanzi et al. 2021)
Policymakers should focus on key variables such as carbon emissions, GDP, and health expenditures when formulating decisions. These factors play a significant role in influencing health risks and outcomes and must be addressed to effectively mitigate related challenges.	Economic	(Al-Ahmadi et al. 2022)
Efforts must be timely and focused on reducing the negative impacts of privatized healthcare. Demonstrating political will and translating it into concrete action are key to overcoming shortcomings in public healthcare, including gaps in quality, patient safety, and clinical performance.	Economic	(Rahman et al. 2020)
Adequate financial support is critical to ensuring the successful implementation of electronic health record systems, regardless of the project's scale.	Economic	(Alzghaibi et al. 2022)
The government must work towards developing a sustainable healthcare system by strengthening the health workforce, promoting decentralization, and ensuring the efficient use of resources through effective stewardship, good governance, and accountability.	Economic	(Alshahrani et al. 2023)
The healthcare system in Saudi Arabia requires comprehensive reform, with particular emphasis on strengthening primary healthcare.	healthcare services	(Al Asmri et al. 2020)
There must be a conscious effort to avoid the framework and strategies focusing on measuring performance regarding transformation, as attention should be placed instead on the health system and health status level.	healthcare services	(Al-Ghamdi et al. 2023)
The Triple Aim model is considered applicable to the Saudi health system, as experts agree on the relevance and suitability of its measures within this framework.	healthcare services	(Al Jasser et al. 2020)
Strengthening collaboration with national and international public and private health organizations can enhance the flow of information available to the Ministry of Health, thereby improving its capacity to develop effective solutions for healthcare institutions. The Ministry should remain committed to advancing the overall well-being and sustainability of the health sector.	healthcare services	(Jimenez et al. 2020)
Decision-makers need to consider several factors when designing PHC policies. For instance, PHC accreditation needs to be prioritized given its positive correlation with service provision and health workers' availability. PHC 24 × 7 operation also needs consideration in rural areas due to the high dependency on PHCCs. Finally, there is a substantial need for improvements in e-health.	healthcare services	(Al Saffer et al. 2021)
Further efforts in the field are required to strengthen medication management systems and prevent incidents that may harm patients.	healthcare services	(Almalki et al. 2021)
Recommended to increase the knowledge and awareness level of the general population through public campaigns and awareness videos on social media applications.	healthcare services	(Almulhim et al. 2021)
Implementation of all MOC interventions will streamline the Saudi healthcare system to embrace the Kingdom's “Vision 2030”. Additionally, the Kingdom must encourage intervention both inside and outside the health system to minimize accidents and the primary and secondary prevention of non-communicable diseases. Also, implement systematic assessments of population needs and health system efficiency to optimize resource distribution and provide the results that people require.	healthcare services	(Chowdhury et al. 2021)
Enabling patient access to their EHR would help promote self-management, a core attribute of effective NCD management. There is also a need for enhanced capacity to support improving patient's nutrition and physical activity.	healthcare services	(Hazazi et al. 2022)
Efficiency in resource utilization and policy planning of resource allocation should consider the different demographic and socioeconomic factors as well as disease.	healthcare services	(Alatawi et al. 2022)
Knowledge of medications could be more inclined to self-medicate as opposed to seeking professional advice from a physician	healthcare services	(Al-Ghamdi et al. 2020)
Recommend enhancing more awareness about hospital efficiency and effective resource utilization for performing appropriate tasks among the stakeholders in the health system of the KSA.	healthcare services	(Alatawi et al. 2020)
KSA needs to increase the adoption of HIS and expand its health workforce and facilities, especially in rural areas.	healthcare services	(Young et al. 2021)
Policy should leverage on this behavior response by introducing insurance packages that share premiums with citizens to incentivize utilization	Health Insurance	(Al-Hanawi et al. 2020)
The type of usual source of healthcare and satisfaction with the current healthcare services were found to have a significant impact on the willingness to pay for NHI.	Health Insurance	(Alharbi et al. 2023)
Desirable attributes for the funding model include collaboration across primary, secondary, and tertiary care. Healthcare finance reform aimed at adopting and increasing personal health insurance coverage may play a critical role in extending access to healthcare, eliminating health inequities, enhancing population health, and reducing government spending on healthcare.	Health Insurance	(Hazazi et al. 2022)
The government should restructure the funding of the healthcare sector so that it becomes self-reliant and less dependent on the government budget.	healthcare services	(Sajjad et al. 2020)
The need to be timely and aimed at mitigating the detrimental effects of privatized health care. Political will and the positive action that stems from it are an effective way to combat inadequacies in the public healthcare sector around the provision of quality healthcare services, patient safety, and clinical effectiveness.	policies and regulation	(Rahman et al. 2020)
Medical waste management practices were found to be inadequate, and significant improvements were strongly recommended.	policies and regulation	(Omer et al. 2020)
To be a competitive member in international trade and tourism, there should be an adequate fund allocation for food safety activities. Continuous efforts should be made to manage and decrease foodborne diseases through the integration of research data, food control monitoring, epidemiological investigations, and disease surveillance	policies and regulation	(Alsubaie et al. 2020)
Analyzing existing policies for their positive and negative impacts on the life chances of different groups in the population would be crucial for addressing the social determinants of health and health equity.	policies and regulation	(Eklund Karlsson et al. 2020)
Relevant stakeholders must take additional measures to ensure the fidelity and sustainability of policy implementation.	policies and regulation	(Almubarak et al. 2023)
The appropriate mixture of financing mechanisms should be informed by evidence-based and data-driven approaches that consider relevant stakeholder preferences.	policies and regulation	(Huraysi et al. 2023)

Table 3 presents the distribution of study by different themes. According to Table 3, the majority of population health management practices fall under the theme of healthcare services by 19 studies. Followed by *E*-health (7 studies) and policies and regulations (5 studies). Furthermore, there was no difference in research activities across the themes of health insurance and economy (5 studies).

**Table 3:** Distribution of studies by themes.

Themes	Reference	N (%)
Economy	[10], [12], [13], [14]	4 (10.3)
*E*-Health	[3], [14], [15], [16], [17], [18], [19]	7 (17.9)
Policies and Regulations	[5], [10], [20], [21], [22]	5 (12.8)
Health Insurance	[4], [23], [24], [25]	4 (10.3)
Healthcare Services	[1], [2], [4], [10], [12], [26], [27], [28], [29], [30], [31], [32], [33], [34], [35], [36], [37], [38], [39]	19 (48.7)
Total		39 (100.0)

### 3.1 Economy

The Saudi healthcare system has recently faced issues in providing universal healthcare for its population. This is attributed to rising healthcare expenditures, declining oil revenues, and increasing life expectancy, the burden of disease patterns, and insufficient population management of public health [16]. Rising healthcare expenditure in Saudi Arabia reflects multiple interconnected factors, including the burden of non-communicable diseases associated with lifestyle patterns and dietary habits [17]. Decision-makers must carefully analyze the relationships between key economic indicators - particularly gross domestic product (GDP) and health expenditure- when formulating healthcare policies. Research demonstrates that strategic health investments, when properly allocated relative to GDP, can support efforts to address population health challenges and improve health outcomes. It is important to note that while economic factors do not directly cause health improvements, they represent critical enabling conditions that determine a health system's capacity to implement and sustain population health interventions. These economic considerations are fundamental to population health management because adequate and sustainable financing directly determines the healthcare system's capacity to implement population-wide interventions, maintain infrastructure, and support the workforce needed to manage the health of entire populations rather than individual patients [17]. In fact, health expenditure requires investment, especially with positive relationships between GDP and increased investment of government spending. A forecasting study by Al-Ahmadi [18] examined the relationship between economic indicators and public health outcomes in Saudi Arabia, finding a strong negative correlation between health issues and both GDP and health expenditure. In other words, more spending on healthcare and a higher GDP were linked to decreased health problems. While this relationship is correlational rather than causal, it suggests that higher GDP and increased healthcare financing are associated with improved public health outcomes. Higher GDP is one of several factors that can contribute to better health outcomes by enabling greater investment in healthcare infrastructure and services. Moreover, a study by Alzghaibi et al. [19] examined the role of economic resources in the implementation of electronic health record (EHR) systems in Saudi Arabia. The findings indicated that adequate financial resources are a key enabling factor for successful EHR implementation, supporting infrastructure development, system selection, training, and technical support. The study also highlighted operational management challenges related to coordinating large-scale digital implementation processes, including system deployment, workflow integration, and ongoing system maintenance. In this context, cost-related challenges refer specifically to the high initial investment and continuing operational costs associated with implementing and sustaining EHR systems. These findings underscore the importance of economic capacity in supporting e-health infrastructure essential for effective PHM [19]. However, the study suggested that effective implementation of e-health technologies, particularly EHR systems, can contribute to addressing several operational and implementation challenges, although not resolving them entirely. The findings emphasized the role of EHR systems in improving information availability, supporting coordination across healthcare services, and facilitating data use for planning and management purposes. By strengthening data infrastructure and system integration, e-health technologies support more efficient organization of healthcare services.

These improvements are directly relevant to PHM, as effective PHM relies on robust health information systems for population-level data collection, care coordination, and informed resource planning. In this context, e-health technologies provide a foundational digital infrastructure that supports the shift towards more proactive and population-focused health management. [19]. In conclusion, the economic status and financial capacity of Saudi Arabia's healthcare system are fundamental determinants of PHM implementation success. Adequate and sustainable financial resources are essential for building the comprehensive infrastructure required for population health management, including data systems (such as EHRs), primary care networks, preventive service programs, and health promotion initiatives. Economic stability also enables the healthcare system to maintain the workforce capacity, training programs, and quality improvement mechanisms needed to manage the health of entire populations rather than simply treating individual patients. Without sufficient economic investment, the healthcare system cannot implement or sustain the population-wide interventions, integrated care models, and data-driven decision-making that are central to effective PHM [16], [17], [18], [19]. Therefore, addressing the economic challenges identified in this theme is a prerequisite for advancing PHM in Saudi Arabia.

### 3.2 *E*-health

Electronic health (e-Health) combines communication technologies and information for the flow of medical information in healthcare environments [20]. Seven studies in this review examined the implementation and adoption of e-health technologies in Saudi Arabia, reflecting growing research interest in digital health solutions. Moreover, the disruption caused by COVID-19 and its consequences on healthcare services resulted in a noticeable growth in the adoption of digital health technologies in Saudi Arabia [21]. In regards to adapting these new technologies, the private sector reports higher levels in comparison to the governmental sector [8].

A critical factor influencing e-health effectiveness is population awareness and digital literacy. A study by Alhodaib and Alanzi [21] examined the growing reliance on digital health services from the perspectives of both policymakers and the Saudi population. The findings of this study illustrated that providing and establishing new technologies in digital health will not serve the primary purpose of these technologies if the public cannot utilize these features easily. Thus, policymakers must consider the population's factors to obtain the best outcome from digital health services. This conclusion is supported by Al-Rayes, AlOfi [8], whose study highlighted the need to enhance the 10.13039/501100004726Ministry of Health (MOH) portal applications to better align with the needs of the Saudi population, particularly in terms of design and usability. Moreover, the majority of respondents in the same study were unaware of, and had never used, the tools provided through the Ministry of Health (MOH) portal. Similarly, a related study examining the knowledge, satisfaction, and barriers to using the *Seha* application reported a low level of utilization. This limited use was attributed to a lack of awareness regarding the app's features and potential benefits [22]. In contrast, the population's satisfaction is reported alongside the fact that the majority of the participants can reflect on the potential benefits of using the application [23]. These findings underscore that e-health technologies are critical enablers of PHM, facilitating data collection, patient monitoring, care coordination, and population-level health surveillance. However, their effectiveness depends heavily on population awareness, digital literacy, and user-centered design—factors that must be addressed to fully realize the potential of digital health in PHM strategies.

### 3.3 Policies and regulations

Historically, the Saudi government provided healthcare services to the general population through the public system. However, in 1999, the government implemented a policy shift that extended private healthcare provision to specific population segments, particularly non-Saudi workers and their dependents, while maintaining public provision for Saudi citizens [16]. Effective policies and regulations are essential infrastructure for population health management, as they establish the legal and operational frameworks within which population-level interventions can be implemented. Among the policies in the aspect of population health management, Several weaknesses were recorded, which included in the content of policies and regulations, such as inadequate legislation, lack of coherence between government entities, increase in imports, lack of explained terminology, weakness of implementing the policies, compliance, and stakeholders' engagement [24], [25], [26]. Relatedly, a study was conducted to analyze the equity dimensions of public health. Policies in the Ministry of Health highlighted equity gaps in the content of policies and strategies. Moreover, analyzing the existing policies in-depth to measure their impact on positive or negative population life changes would be essential for addressing the social determinants of health [24]. Nonetheless, the analysis of several policies revealed a lack of policy evaluation measures that must be implemented in the design phase of the policy to overcome the challenges of policy sustainability and confusion [26]. Furthermore, a study employed differences in policy ranking and feasibility options among 513 participants from all 13 administration regions. The results indicated that most participants preferred financing healthcare by reallocating public financial resources from selected non-health government sectors (such as defense, social protection, and prevention) towards the healthcare sector, rather than increasing overall government expenditure. They also supported policies that impose strict penalties for health-related issues, including waste management. However, notable variations were observed among participants in the ranking of specific policy options. These policy gaps directly affect population health by limiting equitable access to healthcare services and the system's ability to address social determinants of health. Consequently, weak policy coherence and implementation may exacerbate health inequities and hinder population health outcomes. Furthermore, the findings underscored that tax-based policies were perceived as the most feasible approach for generating healthcare funding [27]. Effective policies and regulations form the essential governance framework for PHM implementation. The identified gaps in policy coherence, stakeholder engagement, and evaluation mechanisms directly impede the healthcare system's ability to implement equitable, population-level health interventions. Strengthening policy frameworks is therefore critical to enabling successful PHM in Saudi Arabia.

### 3.4 Health insurance

Health insurance mechanisms are critical enablers of population health management because they determine financial access to healthcare services for entire populations. By expanding insurance coverage and designing benefit packages that prioritize preventive care and chronic disease management, insurance systems can facilitate the population-level health interventions that are central to population health management. A study conducted by Al-Hanawi, Mwale and Kamninga [28], highlighted the need to expand population access to health insurance in Saudi Arabia. This issue cannot be examined independently of the factors linked to health insurance—such as the higher probability of individuals seeking routine medical check-ups—which presents an added challenge for policymakers to consider. This finding is supported by a study conducted by Hazazi, Wilson and Larkin [29], which reported the unsustainability of the current health funding model due to the growing financial burden on the government. Consequently, the evolving landscape of the healthcare sector underscores the necessity of introducing personal health insurance schemes to complement government healthcare spending. In alignment with these findings, Alharbi [30] conducted a study to assess the willingness of the Saudi population to pay for a National Health Insurance (NHI) system. The results provided valuable insights for policymakers, revealing that a majority of participants expressed willingness to contribute to an NHI scheme, with a median payment of 100 SAR per month. Factors associated with greater willingness to pay included prior utilization of public health services and higher satisfaction with the quality of care received. Additionally, the study found that individuals aged 50 years and older were less willing to pay compared to those in the 30–39-year age group. Health insurance mechanisms are critical enablers of PHM, as they determine financial access to healthcare services for entire populations. Expanding insurance coverage and designing benefit packages that prioritize preventive care and chronic disease management can facilitate the population-level health interventions that are central to effective PHM implementation.

### 3.5 Healthcare services

The healthcare services in Saudi Arabia are evolving and facing many issues in terms of providing access to the population [4], [31]. Particularly in primary healthcare, it has found major areas of improvement, such as the scope of services, infrastructure, financing, and increased capacity, and it has been struggling with the transition towards the 2030 vision of Saudi Arabia [4]. Evaluating healthcare services within a PHM framework requires appropriate measurement tools and expertise. The Triple Aim model—a widely recognized framework in population health management that focuses on improving population health, enhancing patient experience of care, and reducing per capita costs—represents an important approach for assessing healthcare system performance from a population perspective [32]. However, a Delphi study by Al Jasser and Almoajel [32] that attempted to assess the applicability of the Triple Aim framework in the Saudi healthcare context encountered significant challenges. The study involved expert participants from various healthcare sectors, but the majority were non-clinical professionals (administrators, policymakers, and managers rather than physicians or clinical practitioners). This limitation in clinical expertise among the expert panel made it difficult to effectively apply the Triple Aim framework's clinical and population health metrics to evaluate healthcare service quality and outcomes. This finding highlights a broader challenge in Saudi Arabia's healthcare system: the need for multidisciplinary expertise that combines clinical knowledge with management and policy perspectives to effectively implement and evaluate PHM initiatives. In terms of accessibility, a study conducted in different regions in Saudi Arabia found that 46.1% of the population faced difficulties in reaching hospitals, in addition, 45.2% found that the healthcare services in health centers were inefficient, and the lack of insurance leads to self-medication rather than going to health centers [33]. For instance, 91 public hospitals were included in the assessment of technical efficiency. Results revealed that among these hospitals, 75.8% were technically inefficient. This indicates that these hospitals could reduce their resource inputs (including staff, equipment, budget, and operational resources) by 24% while maintaining the same level of healthcare service provision. Moreover, the causes of inefficiency were identified as the overutilization of resources and the insufficient production of health services [34]. Similarly, a study conducted among public hospitals under the Ministry of Health's administration revealed that external factors—including the regulatory environment, geographic location, local market competition, and socioeconomic characteristics of the area served—have a major influence on hospital efficiency [35]. The study also found that technical efficiency scores are significantly associated with the population volume served by each hospital, where “population volume” refers to the size of the catchment population that relies on the hospital for healthcare services [35]. This association suggests that hospitals serving larger populations may experience different efficiency dynamics due to economies of scale, higher patient volumes that enable better resource utilization, and different patterns of healthcare demand. Understanding these external influences and population-related factors is essential for PHM planning, as it indicates that efficiency improvement strategies must be tailored to each hospital's specific context and the population health needs of the communities they serve. In this context, strategic planning and effective management are essential requirements for the healthcare system to develop evidence-based policies and programs, improve healthcare service delivery, and systematically measure the impact of interventions on population health outcomes [36]. Therefore, building relationships internationally in the private and public health sectors is essential to improving the system [37]. At the national level, the primary health care services provide care to 0.74 per 10,000 of the population. However, the distribution of healthcare services across regions needs to be optimized. Several doctors and specialties are found in primary healthcare. However, psychiatrists and nutritionists are hard to find. Nonetheless, policymakers need to consider factors including demand, geographic distribution, and the use of e-health [38]. The healthcare services aim to provide optimal care for the population. However, evidence suggests that medical errors remain a persistent challenge within hospital settings, particularly medication-related errors such as prescribing, dispensing, and administration errors [39]. Moreover, the healthcare system faces challenges related to healthcare service utilization patterns. A study conducted in Saudi Arabia revealed that 49.6% of participants preferred visiting the emergency department rather than primary healthcare centers when experiencing acute or urgent medical problems, although more than 90% reported primary healthcare centers as their usual source of care [40].

This apparent contradiction reflects differences between general healthcare utilization patterns and care-seeking behavior in acute situations, where patients may prioritize immediate access to services over continuity of care. Participants reported preferring emergency departments due to quicker access to medical care, easier access without prior appointments, same-day availability, and the perception of more comprehensive examinations [40]. This preference pattern poses a significant challenge for PHM implementation, as it contributes to emergency department overcrowding, inefficient resource utilization, and missed opportunities for preventive care and chronic disease management in primary care settings.

The population of Saudi Arabia is rising and is expected to reach 39.5 million, by 2030. Primary healthcare services continue to be insufficient. In addition, Secondary, tertiary, and specialized hospitals, along with their associated services, are scattered geographically among the main regions [6], [41]. The point of view of the Ministry of Health's leaders revealed that the healthcare leaders lack the necessary capacity to effectively utilize resources to meet the growing demand for healthcare services, and many measurements lack clear objectives and goals, in addition to poor strategic planning, which makes it a major obstacle to optimal performance [42]. Relatedly, the lack of centralized decision-making in the health governmental authority caused major barriers to efficiency in public health, and the major challenge with population health management is population awareness [43]. The efficiency, accessibility, quality, and distribution of healthcare services are fundamental determinants of PHM success, as they constitute the delivery infrastructure through which population-level health strategies are operationalized. Addressing the identified challenges in service capacity, geographic distribution, technical efficiency, and population awareness is essential for translating PHM principles into measurable health improvements across the Saudi population.

The undeniable successes and changes happening in Saudi Arabia due to the Saudi 2030 vision are important indicators that can be utilized to expect the future achievements of the country. As one of the main aspects of the 2030 vision is the healthcare system, it was made mandatory to study the current status of the system from the aspects that can be linked to the Saudi 2030 vision. In this literature review, the healthcare aspects were studied from the perspective of population management in Saudi Arabia. Healthcare services, policies and regulations, economics, e-health, and health insurance were the headlines looked for, which resulted in 34 studies. The healthcare services in Saudi Arabia are reported to be facing many obstacles that have to be addressed to provide the population with optimal services that can follow the revolutionary vision of Saudi Arabia [4], [33]. Difficulty in reaching the hospitals, the preference for going to ED rather than the PHC, overuse of resources, lack of suitable manpower in the leadership roles, and population awareness are reported to be the main challenges ahead of serving the best healthcare [4], [33], [40]. While secondary, tertiary, and specialized hospitals are present throughout Saudi Arabia, their geographic distribution is concentrated in major urban regions, creating accessibility challenges and rural areas. Additionally, primary healthcare services remain insufficient relative to population needs, particularly given the projected population growth to 39.5 million by 2030. Therefore, accessibility to appropriate healthcare services remains a significant concern for substantial portions of the Saudi population, despite the physical availability of facilities in some regions [6], [40]. This fact can be linked to the next obstacle, where the population prefers the ED over the PHCs which disturbs the healthcare system and becomes a barrier ahead of the reforms [40]. The challenge of suboptimal primary healthcare utilization is closely linked to patient engagement, which is a critical component of effective PHM. Al Ghamdi's comprehensive research on primary healthcare engagement in Saudi Arabia identified multiple barriers to appropriate patient engagement, including limited health literacy, cultural factors, accessibility issues, and gaps in patient-provider communication [44]. These barriers not only affect individual care-seeking behavior but also impede population-level health management efforts. The COVID-19 pandemic further highlighted these engagement challenges while simultaneously demonstrating the potential for digital health solutions to enhance patient engagement with primary healthcare services [45]. Addressing these patient engagement barriers is essential for successful PHM implementation, as population health management fundamentally depends on active participation and engagement of the population in preventive care, chronic disease management, and health promotion activities. Moreover, the reforms are not going to be realistic goals if the people who are in charge are incapable of sharing the same perspective, efficacy, and commitment to the Saudi 2030 vision. While the lack of population awareness is a well-reported fact [9], this fact can be attributed to the poor strategic planning of the leaders and their inability to utilize the resources to deliver the best performance in the healthcare services [6], [41]. Therefore, the healthcare services are yet to meet by population's needs and expectations. The healthcare policies and regulations in Saudi Arabia have gone through many transitions throughout the years to adopt the policies and regulations that match the governmental reforms alongside the population's needs [16]. However, the content of existing policies and regulations reflects several challenges, including inadequate legislation, lack of coordination among government entities, increased dependence on imports, and unclear or undefined terminology, resulted in weakness in implementing the policies, lack of compliance, and stakeholders' engagement, as these issues were reported as the main factors that affected the establishment of the best policies ad regulations [24], [26], [46]. The population does not engage in the process directly; however, the results of these decisions are reflected in the population, so any disruption within the policies and regulations formation phase will cost the policymakers the population's confidence. Hence, policymakers should consider public opinions in a manner that avoids bias in the regulatory process. Moreover, if the policymakers are not able to control the external engagements of stakeholders or cannot implement the policies and regulations with a steady hand, the population will not benefit from any new policy or regulation. As a result of the policies and regulations, the effect on the economy is noticeable in the issues of providing universal healthcare for the Saudi population. With rising healthcare expenditure, declining oil revenues, increased life expectancy, the burden of disease patterns, and insufficient population management of public health are reported to be causal factors of this dilemma [16]. The effect of these issues on the GDP is a well-reported fact [18]. Moreover, public health issues are to be solved with the increased dependency on GDP. However, this solution is contraindicated with the Saudi 2030 vision, which is trying to overcome these issues by providing a structured transformation in healthcare. The healthcare transformation is shifting to be a need more than just an improvement in the field [37]. The economic burden reported with the increased life expectancy, the need to make the healthcare sector more self-reliant, and the need to design the transformation plan in a way that will fulfill the needs of the population and meet their expectations are reported to be the main objectives related to the population [42]. Hence, healthcare infrastructure, adequate staff training, and improved accessibility to healthcare services in non-urban area centers must be primary goals to be achieved to deliver the transformation smoothly [17]. A main defense line utilized by the transformation in healthcare is electronic health (e-health). The concept of e-health is not new; however, its need was raised promptly during the COVID-19 pandemic outbreak [21]. The world was forced to shift to e-health, where the Saudi MOH illustrated high levels of efficacy and ability to utilize the privileges of e-health to serve the Saudi population with the optimum benefits and easiest transitions of healthcare systems into digital health technologies [8]. This transition cannot serve its purpose if the population is not able to use these technologies appropriately [47]. Hence, e-health is mandated to provide the healthcare systems with the desired solutions for the reported burdens. In contrast, without proper population educational campaigns in this regard, e-health is going to be an established burden by itself. The financial burden on the government has highlighted the need for personal healthcare insurance. This need is represented by the unsustainability of the current model in health funding mechanisms [43]. The Saudi population is not against the idea of an NHI system, but the issues reported mainly with the cost of insurance [48]. Therefore, the NHI system may relieve some of the burdens if the population's perspective is taken into consideration.

This study conducted a review of 34 studies related to population health management in Saudi Arabia, analyzing key aspects such as healthcare services, policies, economics, e-health, and health insurance. As a result, it provides significant insights into the existing literature. However, the search was limited to studies published between 2020 and 2024, which may have excluded relevant research conducted before this period. In addition, some relevant studies may have been missed due to the predefined search strategy and inclusion criteria. Another limitation is that while the study examines e-health as a crucial component, the included studies focus only on two specific areas: m-health and EHR, potentially overlooking other important aspects of e-health. Furthermore, although the study identifies population education as a critical challenge, it does not offer an in-depth analysis or recommendations for addressing this issue. Future research should incorporate empirical studies or case analyses to further validate and expand upon these findings.

## 4 Conclusion

This narrative review illustrates that among healthcare services, policies and regulations, economics, e-health, and health insurance in Saudi Arabia, population engagement and education remain the primary challenges to implement an effective PHM. The transformation plan towards Vision 2030 is promising. Yet, the success depends on the strengthening of the population-centered strategies improving health literacy, and enhancing system integration. Future direction should focus on workforce capacity building, leveraging digital health platform and conducting national campaigns to enable people participate effectively in PHM initiatives with continued support to sustain improvement of the health outcomes.

## Ethics and other permissions

Not applicable.

## CRediT authorship contribution statement

**Khalid Alkhurayji:** Writing – original draft. **Abdallah Alsuhaimi:** Writing – review & editing, Supervision. **Sultan Aldakhil:** Writing – review & editing. **Dalal Alshathri:** Writing – review & editing.

## Declaration of competing interest

The authors declare that they have no competing interests.

## Data Availability

All data generated or analysed during this study are included in this published article [and its supplementary information files].
